# *Prevotella copri* is associated with carboplatin-induced gut toxicity

**DOI:** 10.1038/s41419-019-1963-9

**Published:** 2019-09-26

**Authors:** Chaoheng Yu, Bailing Zhou, Xuyang Xia, Shuang Chen, Yun Deng, Yantai Wang, Lei Wu, Yaomei Tian, Binyan Zhao, Heng Xu, Li Yang

**Affiliations:** State Key Laboratory of Biotherapy and Cancer Center, West China Hospital, Sichuan University and Collaborative Innovation Center of Biotherapy, Chengdu, Sichuan China

**Keywords:** Chemotherapy, Dysbiosis

## Abstract

As a widely used cancer drug, carboplatin often results in serious side effects, such as gut toxicity. In this study, we examined the effects of gut microbiota on mice with carboplatin-induced intestinal mucosal damage. Carboplatin resulted in intestinal mucositis, as indicated by weight loss, diarrhoea, and infiltration of inflammatory cells. It markedly increased the expression of inflammatory cytokines/chemokines in intestine. Carboplatin also altered the diversity and composition of the gut microbiota. A significantly higher abundance of *Prevotella copri* (*P. copri*) was observed in carboplatin-treated mice. Moreover, the content of *P. copri* was positively correlated with the severity of intestinal mucositis. Pretreatment with metronidazole reduced the content of *P. copri* and relieved the intestinal mucosal injury and inflammation that was induced by carboplatin. Further study revealed that supplementation with *P. copri* in carboplatin-treated mice resulted in more severe tissue damage, lower tight junction protein expression and higher cytokine expression, and it enhanced both local and systemic immune responses. These data demonstrated that *P. copri* was involved in the pathological process of carboplatin-induced intestinal mucositis, suggesting a potential attenuation of carboplatin-induced intestinal mucositis by targeting *P. copri*.

## Introduction

The treatment of cancer is still largely based on the ability of chemotherapeutic drugs to eliminate cancer cells, reduce tumour growth, and relieve pain^1^. The antitumour activity of most chemotherapeutic drugs depends on the induction of DNA damage in rapidly proliferating tumour cells, resulting in inadequate DNA repair and apoptosis^2^. For example, platinum drugs form adducts with DNA, which lead to intrastrand and interstrand crosslinks, and anthracyclines inhibit DNA topoisomerases and produce oxygen radicals^1^. Chemotherapy is not specific to tumour cells but can also induce deleterious effects on rapidly dividing cells, such as bone marrow and gut mucosal cells.

Myelosuppression, oral and gastrointestinal toxicities often occur during chemotherapy, leading to a reduction in the dose of chemotherapy or even the termination of treatment. Myelosuppression can be treated with growth factors or supplemented by bone marrow transplantation^3^. However, there is no efficient treatment to reduce chemotherapy-induced oral and gastrointestinal mucosal lesions^4,5^. Epithelial cells of the intestinal mucosa are mainly produced by the continuous proliferation of crypt cells. After proliferation, the cells differentiate and migrate to the villi, where they replace the mature epithelial cells that are shed from the tips^6^. Chemotherapeutic drugs impair mucosal absorption and barrier function by killing crypt stem cells. Symptoms of intestinal mucositis include ulceration, inflammation, nausea, abdominal bloating, abdominal pain and diarrhoea^7^. These symptoms generally peak on the third day after chemotherapy initiation. It has been reported that the incidence of intestinal mucositis in cancer therapy was >40% in patients who received standard chemotherapy and almost 100% in patients who received high doses of therapy^5,8^. Therefore, chemotherapy-induced intestinal mucositis is a non-negligible problem in the treatment of cancer.

Recently, accumulating evidence suggests that the gut microbiota is involved in modulating the efficacy and toxicity of chemotherapeutic drugs^9–12^. The gut microbiota encompasses ~3 × 10^13^ bacteria cells, most of which exhibit commensalism with the host^13^. However, when the intestinal ecology changes, commensal bacteria may expand and acquire pathogenic characteristics. The gut microbiota also plays an important role in host health by regulating the immune response, mucosal barrier function, dietary fibre digestion, vitamin synthesis and metabolism^14,15^. In addition, therapeutic drugs can also have an impact on the gut microbiota. For example, previous studies have reported that 5-fluorouracil (5-FU) treatment reduces the abundance of *Lactobacillus* spp. and *Clostridium* spp. in the jejunum of rat, while irinotecan increases *Enteroccoccus* spp., *Serratia* spp., *Lactobacillus* spp., and *Clostridium* spp.^16,17^. Viaud et al. found that the total number of bacteria in the small intestine of mice did not decrease after 7 days of cyclophosphamide treatment, but the abundance of *Lactobacilli* and *Enterococci* was reduced^18^. Cyclophosphamide treatment resulted in shortening of the small intestinal villi, discontinuity of the intestinal barrier, focal accumulation of inflammatory cells. Furthermore, specific Gram-positive bacteria (*Lactobacillus johnsonii* and *Enterococcus hirae*) were required for mediating the cyclophosphamide-driven accumulation of Th1 and Th17 cell responses^18^. CTLA4 antagonism has been reported to induce T cell-mediated intestinal mucositis, which was associated with alterations of the gut microbiota^19^.

In this study, we chose carboplatin, a widely used chemotherapy drug, to induce intestinal mucositis. We hypothesised that carboplatin could cause changes in the gut microbiota, and these changes were associated with the development of intestinal mucositis. Our aim was to characterise these changes and examine the effects of the gut microbiota on mucositis induced by carboplatin.

## Materials and methods

### Mice

Female C57BL/6 mice (6–8 weeks old) were obtained from Beijing Vital River Laboratory Animal Technology Co., Ltd. (Beijing, China). They were maintained under specific pathogen-free conditions on 12-h light/dark cycles at 22 ± 2 °C. All animal experiments were conducted in accordance with the guidelines approved by the Animal Care Committee of Sichuan University (Sichuan, China).

### Bacterial culture

*Prevotella copri* (DSM 18205) were purchased from Beijing Biobw Biological Technology Co. Ltd. (Beijing, China). The bacteria were cultured in PYG liquid medium (Qingdao hopebio Technology Co. Ltd., Qingdao, China) under anaerobic conditions. Bacterial suspensions were washed with sterile PBS, centrifuged and resuspended in PBS to an OD600 = 1, which corresponds approximately to 1 × 10^9^ colony forming units (CFU)/ml.

### Animal experiments

We performed three experiments to study the relationship between the toxicity induced by carboplatin and the gut microbiota. In the first experiment, female C57BL/6 mice (6–8 weeks old) were treated with a single 100 mg/kg intraperitoneal (i.p.) dose of carboplatin (Selleck, Shanghai, China) for 7 days. Control mice received sterile PBS only. In the second experiment, C57BL/6 female mice were randomly divided into four groups (five mice per group). The Met group and Met + Car group mice were treated with metronidazole (1 g/L) in their drinking water on day −7 for 2 weeks. After 1 week (day 0), Car group and Met + Car group mice were injected i.p. with 100 mg/kg of carboplatin, while the other two groups (Met group and Con group) were treated with sterile PBS. The experiment ended at day 7. In the third experiment, mice in the Met + Car and Met + Car + *P*. *copri* group were treated with metronidazole on day −7 for 5 days, followed by 2 days of drug withdrawal. On day 0, three groups of mice (Car, Car + *P*. *copri*, Met + Car and Met + Car + *P*. *copri* group) were injected with 100 mg/kg of carboplatin, *P*. *copri*, Car + *P*. *copri* and Met + Car + *P*. *copri* group mice were simultaneously gavaged with 1 × 10^8^ CFU of *P*. *copri*. Control group mice were gavaged with sterile PBS. All the mice were sacrificed at day 7. The weight of the mice in all three experiments was recorded daily.

### Faecal sample collection and DNA extraction

According to different animal experimental protocols, faecal samples were collected from mice on day −7, day 0, or day 7 and immediately stored at −80 °C until use. Bacterial DNA was extracted using the Stool DNA Isolation Kit (Foregene Co., Ltd., Sichuan, China) according to the manufacturer’s instructions. Samples were stored at −80 °C.

### 16S sequencing and bioinformatics

The V4 region of bacterial 16S rDNA was amplified at Beijing Novogene Technology Co., Ltd., and sequenced on an Illumina MiSeq with 500-bp paired-end reads. The Illumina Sequence data were analysed using Mothur version 1.35.1^20^, closely following their MiSeq SOP^20^. Forward and reverse read MiSeq data were assembled into paired-read contigs, and then, all resulting contigs that were shorter than 240 bp, longer than 260 bp, contained any ambiguous bases, or contained homopolymers longer than eight bases were removed. Sequences were aligned using the Silva reference alignment as a template, and potentially chimeric sequences were eliminated using the Uchime algorithm^21^ incorporated into Mothur. A preclustering step (diffs = 2) was carried out to reduce the impact of sequencing errors, and any reads that were derived from chloroplasts, mitochondria, Eukarya, or Archaea were also dislodged. Sequences with a distance-based similarity of ≥97% were clustered into operational taxonomic units (OTUs) using the furthest-neighbour algorithm. Each OTU was given a taxonomic classification using the Greengenes reference database. To measure bacterial diversity, the dataset was first random subsampled to 44,089 sequences/sample to ensure an equal sequencing depth among each of the samples. Following this step, the alpha diversity (i.e., ACE and Chao1) indices for each subsample was calculated in Mothur. The unpaired, two-tailed *t* test was used to calculate differences between means (GraphPad Prism version 6.01; GraphPad Software). Feature selection of the intestinal microbial composition was performed on OTUs with an average abundance >0.01% in each mouse group^22^. Furthermore, a distinct clustering effect was observed upon performing principal coordinate analysis on unweighted UniFrac distances^23^.

The linear discriminant analysis (LDA) with effect size (LEfSe) method of analysis^6^ first compared abundances of all bacterial clades (in this case between Day 0 and Day 7) using the Kruskal–Wallis test at a pre-defined *α* of 0.05. Significantly different vectors resulting from comparisons of abundances between groups were used as input for LDA, which produced an effect size. The primary advantage of LEfSe over traditional statistical tests was that an effect size was produced in addition to a *p*-value. This feature allowed sorting of the results of multiple tests by the magnitude of the difference between groups.

### 16S rDNA quantitative PCR

16S rDNA quantitative real-time PCR (qPCR) was performed to quantify bacterial 16S rDNA gene abundance in faeces. Primer sets targeting rDNA genes of *Prevotella* at the genus level were used and included *P. copri* (F: CCGGACTCCTGCCCCTGCAA, R: GTTGCGCCAGGCACTGCG AT), *P. melaninogenica* (F: GTGGGATAACCTGCC GAAAG, R: CCCATCCATTACCGATAAATCTTTA), and *P. ruminicola* (F: GAAA GTCGGATTAATGCTCTATGTTG, R: CATCCTATAGCGGTAAACCTTTGG), and Universal 16S Primers (F: ACTCCTACGGGAGGCAGCAGT, R: ATTACCGCGGCT GCTGGC). qPCR was performed on a CFX96 Real-Time System (Bio-Rad) with the following cycling conditions: 95 °C for 10 min, 35 cycles at 95 °C for 15 s, 60 °C for 60 s, and 72 °C for 30 s.

### Immunohistochemistry and immunohistofluorescence

For histological analyses, the whole small intestine (duodenum, jejunum and ileum) and colon samples were removed and fixed in 4% PFA followed by paraffin embedding. Sections (4 μm) were stained with haematoxylin and eosin (H&E) and scored by a pathologist. For the histological semiquantitative analyses, villus atrophy, necrosis, and inflammatory cell infiltration were scored for each section as follows: 0 (no damage), 1 (mild damage), 2 (moderate damage) and 3 (severe damage). The length of the villus was measured in 10 intact and well-oriented villi using ImageJ software (NIH, Bethesda, MD). For immunohistofluorescence, the sections were dewaxed in xylene and rehydrated in a decreasing alcohol gradient. After washing with PBS, the sections were placed in citrate antigen retrieval solution (10 mM, pH 6.0) and heated in a pressure cooker for 3 min. The sections were cooled to room temperature, washed in PBS, and blocked with 3% BSA for 1 h at room temperature. Antibody (PE anti-mouse CD11b or FITC anti-mouse CD3, BD Biosciences) was applied for 1 h at room temperature at a dilution of 1:100 followed by nuclear counterstaining with DAPI.

### Flow cytometric analysis

Monoclonal antibodies against mouse CD3e, CD4, IFN-γ, IL-17A, CD11b, CD11c, F4/80, and CD206 were purchased from BD Biosciences. Mice were sacrificed at the end of the experiments. Spleens and mesenteric lymph nodes (MLN) were moved and smashed in a cell strainer (75-μm Nylon; BD Falcon). Cell suspensions were treated with red blood cell lysis buffer (Beyotime Biotechnology, Shanghai) for 5 min at room temperature. After washing three times, the cells were finally suspended in PBS. For surface staining, cell suspensions were sequentially incubated with various combinations of antibodies for 30 min at 4 °C. To stain the intracellular IL-17A and IFN-γ, cells were fixed with 2% neutral paraformaldehyde after surface marker staining, treated with 0.5% Triton-100 (diluted in PBS) for 20 min at 4 °C, washed again, and incubated with anti-mouse IL-17A or anti-mouse IFN-γ. Cell events were acquired with a flow cytometer (FACSCalibur; BD Biosciences), and data were analysed using FlowJo software (Tree Star).

### Cell culture and stimulation with *P. copri* in vitro

Macrophage RAW264.7 cells and intestinal epithelial IEC6 cells were cultured in DMEM (GIBCO, USA) supplemented with 10% foetal bovine serum (GIBCO, USA) at 37 °C in 5% CO_2_. For the pro-inflammatory assay of *P. copri*, RAW264.7 or IEC6 cells were plated at a density of 5 × 10^5^ per well in six-well plates and then stimulated for 4 h with medium alone or carboplatin (10 μM) or *P. copri* (multiplicity of infection = 10) in the presence or absence of carboplatin. After incubation, the cells were collected and evaluated for gene expression analysis.

### Gene expression analysis

Total RNA from cells (RAW264.7 and IEC6 treated as described above) or ileum segments of mice were extracted using RNAiso Plus (Takara, Japan) and then quantified with a Nano Drop ND-1000 spectrophotometer (Thermo Scientific). cDNA was synthesised using the PrimeScript RT Reagent Kit with gDNA Eraser (Takara, Japan) according to the manufacturer’s instructions. Transcript levels of IFN-γ, IL-1β, IL-6, MCP-1, claudin-1, occludin, ZO-1 and β-actin were analysed using SYBR Green PCR Master Mix (Invitrogen) on a CFX96 Real-Time System (Bio-Rad). Relative expression was calculated using the ΔCt method. Gene-specific primer sequences were as follows: IFN-γ (F: ACAGCAAGGCGAAAAAGGATG, R: TGGTGGACCACTCGGATGA); IL-1β (F: ATCTCGCAGCAGCACATCAA, R: ACGGGAAAGACACAGGTAGC); IL-6 (F: CCAGTTGCCTTCTTGGGACT, R: GTCTCCTCTCCGGACTTGTG); MCP-1 (F: GATGCAGTTAACGCCCCACT, R: CCCATTCCTTCTTGGGGTCA); claudin-1 (F: TGCCCCAGTGGAAGATTTACT, R: CTTTGCGAAACGCAGGACAT); occludin (F: TGAAAGTCCACCTCCTTACAGA, R: CCGGATAAAAAGAGTACGCTGG); ZO-1 (F: GCCGCTAAGAGCACAGCAA, R: GCCCTCCTTTTAACACATCAGA) and β-actin (F: TATGGAATCCTGTGGCATC, R: GTGTTGGCATAGAGGTCTT).

### Statistical analysis

Data were analysed using GraphPad Prism 6 (GraphPad, La Jolla, CA, USA). All values are depicted as the means ± SEM unless otherwise noted. Statistical comparisons were performed using unpaired two-way analysis of variance (ANOVA) or the *t* test with Bonferroni correction (for parametric data) as needed. *p* < 0.05 was considered statistically significant.

## Results

### Carboplatin induces intestinal mucositis in mice

**Fig. 1:**
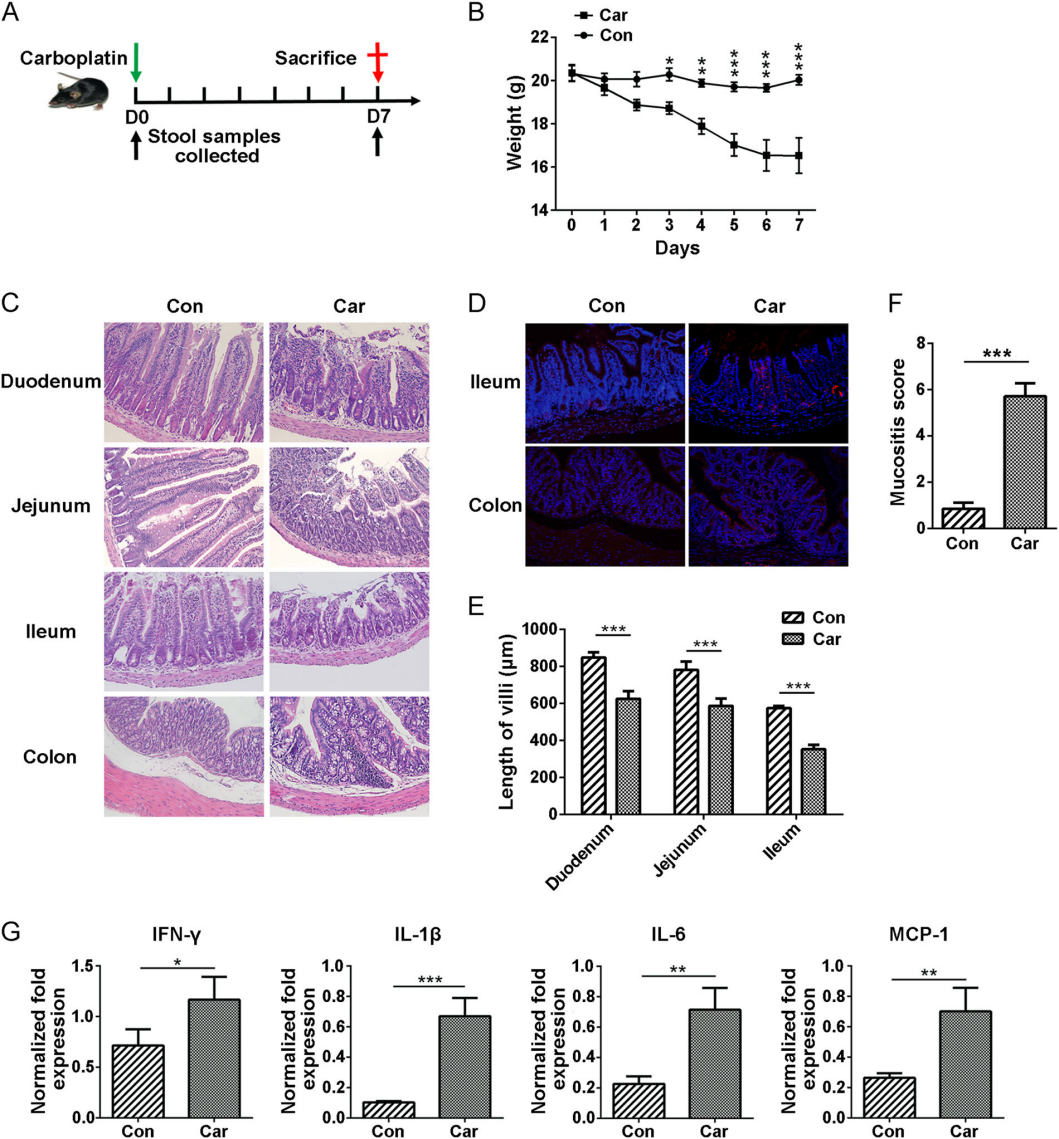
Carboplatin treatment induces severe intestinal mucositis. **a** Schematic of carboplatin administration and sample collection. C57BL/6 mice were treated intraperitoneally with carboplatin or saline on day 0 and then were sacrificed on day 7. The stools of mice were collected before and after the treatment. **b** Body weight changes in control mice and carboplatin-treated mice from day 0 to day 7. **c** H&E staining of duodenum, jejunum, ileum and colon tissues. (magnification, ×200). **d** Immunofluorescence staining of ileum and colon tissues. Blue represents the nucleus, and red represents CD11b^+^ cells (magnification, ×200). **e** Villus length of the duodenum, jejunum and ileum tissues. **f** Histological mucositis scores for the small intestine and colon. **g** Expression levels of IFN-γ, IL-1β, IL-6 and MCP-1 in ileum tissues of mice after treatment with carboplatin or saline. Data are presented as the mean ± SEM. *p *<* 0.05, **p *<* 0.01, ***p *<* 0.001

To investigate the gut toxicity of carboplatin, mice were treated with a single dose of carboplatin (100 mg/kg, i.p.) for 7 days (Fig. 1a). Carboplatin caused diarrhoea in mice, and their weights decreased beginning on day 2. In contrast to the controls, weight loss following carboplatin treatment was most severe on day 7 (Fig. 1b). Compared with the control group, the carboplatin-treated mice exhibited obvious villous atrophy and intestinal epithelial damage in the small intestine tissues, as shown in Fig. 1c and e. In addition, obvious inflammatory cell infiltration was observed in colon tissues of carboplatin-treated mice (Fig. 1c). Immunofluorescence staining of the ileum and the colon showed an aberrant accumulation of CD11b^+^ myeloid cells in the carboplatin-treated mice but not in the control mice (Fig. 1d), while no difference in CD3^+^ T cells was detected (Fig. S1). The intestinal mucositis score was markedly increased in the carboplatin group compared with the control group (*p* < 0.001) (Fig. 1f). These results suggest that carboplatin treatment can cause severe intestinal damage, which is accompanied by infiltration of CD11b^+^ myeloid cells in the gut.

To further verify the carboplatin-induced intestinal inflammatory response, the expression of inflammatory markers in ileum tissues was detected using real-time PCR.

We observed that the expression levels of the pro-inflammatory cytokines IFN-γ, IL-1β, IL-6 and IL-17A were significantly elevated in the ileum of carboplatin-treated mice (Figs. 1g and S2). MCP-1 is a monocyte chemokine that regulates the migration and infiltration of monocytes, macrophages, memory T lymphocytes, and natural killer (NK) cells^24,25^. Our results showed that compared with the control group, the expression of MCP-1 was significantly upregulated in the intestinal tissue of mice after treatment with carboplatin (*p* < 0.01) (Fig. 1g), which may increase the migration of monocytes or macrophages to sites of intestinal injury and further promote local inflammatory responses. These data were consistent with an increase in CD11b^+^ myeloid cell infiltration in intestinal tissues.

### Carboplatin induces alterations in macrophage and T cell subsets

**Fig. 2:**
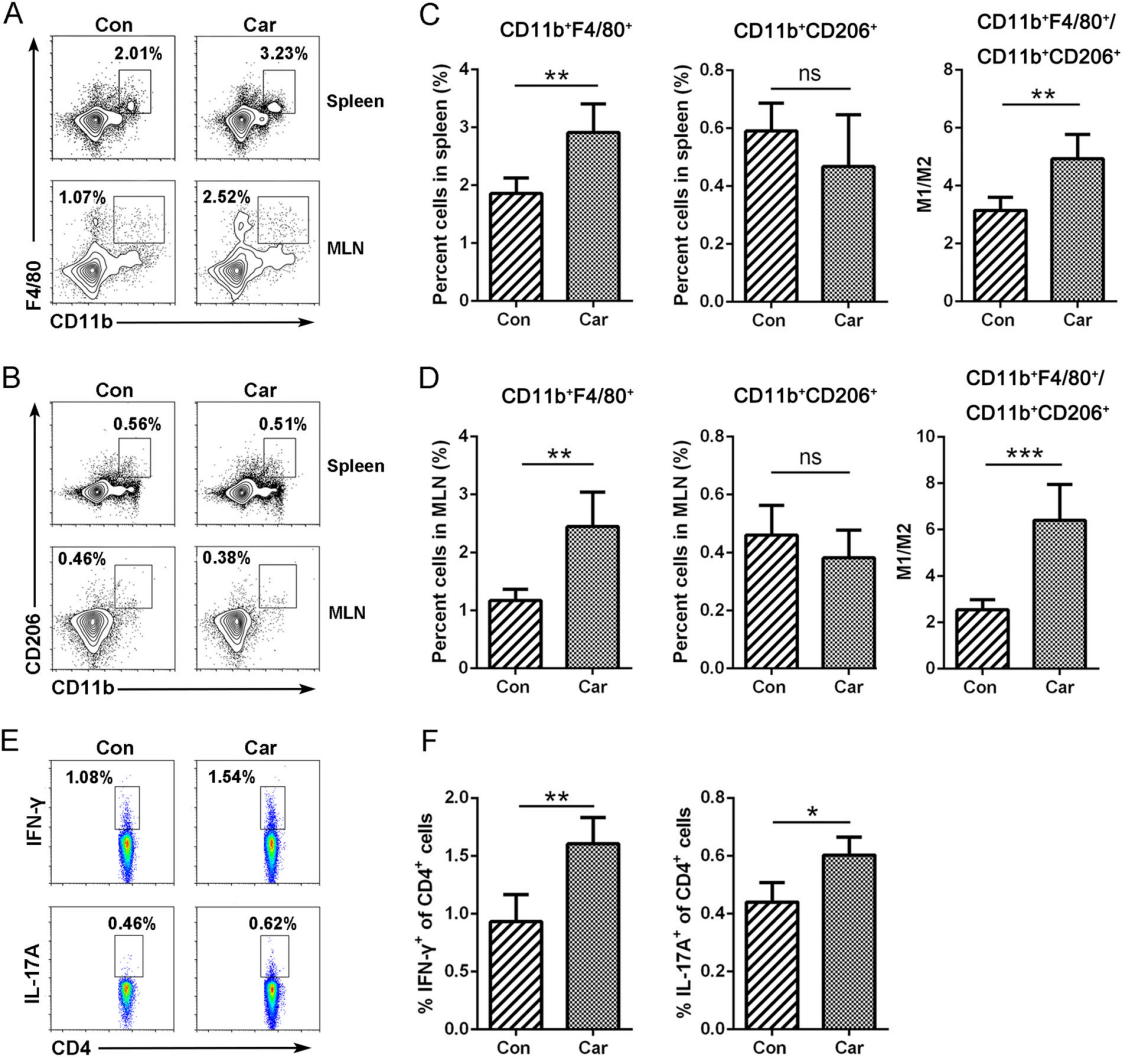
Carboplatin induces changes in macrophage and T cell subsets. Percentages of CD11b^+^F4/80^+^ **a** and CD11b^+^CD206^+^ macrophages **b** in spleens and MLNs of mice treated with carboplatin or saline by flow cytometry. Quantification of CD11b^+^F4/80^+^ and CD11b^+^CD206^+^ macrophages in spleens **c** and MLNs **d**. **e** and **f** Percentages of Th1 and Th17 cells in MLNs. Data are presented as the mean ± SEM. *p *<* 0.05, **p *<* 0.01, ***p *<* 0.001

We noticed that the infiltration of CD11b^+^ mononuclear phagocytes in intestinal tissues was significantly increased in response to carboplatin treatment compared to the control (Fig. 1d), while CD11b^+^ cells could be subdivided into macrophages and dendritic cells (DCs). In tissues, macrophage responses to various signals can acquire specialised functional phenotypes: classical activated macrophages (CD11b^+^F4/80^+^) and alternative activated macrophages (CD11b^+^CD206^+^)^26^. CD11b^+^F4/80^+^ macrophages secrete chemokines and pro-inflammatory cytokines, present antigens, and promote Th1 responses, which are mainly involved in a positive immune response. In contrast, CD11b^+^CD206^+^ macrophages exert immunomodulatory functions by producing inhibitory cytokines, such as IL-10 or TGF-β and downregulating the immune response^27,28^. Treatment with carboplatin caused significantly higher CD11b^+^F4/80^+^ macrophage content (*p* < 0.01) and slightly lower CD11b^+^CD206^+^ macrophage content (*p* > 0.05) in both the spleens and the MLN compared with the control group (Fig. 2a–d). Moreover, the ratio of CD11b^+^F4/80^+^/CD11b^+^CD206^+^ in carboplatin-treated mice was significantly increased (*p* *<* 0.01), indicating that carboplatin facilitated the polarisation of CD11b^+^F4/80^+^ pro-inflammatory macrophages in mice, thereby promoting intestinal inflammation. There were no statistically significant differences in the number of DCs between the carboplatin treatment group and the control group (Fig. S3).

A previous study has reported that cyclophosphamide disrupts the integrity of the intestinal barrier in mice and activates T helper (Th) immune responses^18,29^. Our results showed that carboplatin treatment significantly increased Th1 (CD4^+^/IFN-γ^+^) and Th17 cells (CD4^+^/IL-17A^+^) in the MLNs of mice compared with the control (Fig. 2e and f), whereas no significant difference could be detected in spleens; this suggests that carboplatin mainly triggered local immune responses.

### Carboplatin induces changes in intestinal microbial components

Recent studies have shown that some anticancer drugs can cause alterations in intestinal microbial components, such as irinotecan, cyclophosphamide, and cisplatin, among others^7,30^. Moreover, the gut microbiota is closely associated with the resistance, therapeutic effect or toxicity of anticancer drugs^31^. To determine whether carboplatin had an effect on the gut microbiota in mice, faecal samples were collected before and after treatment for bacterial microbiota profiling using 16S ribosomal DNA (rDNA) sequencing on the Illumina MiSeq platform.

**Fig. 3:**
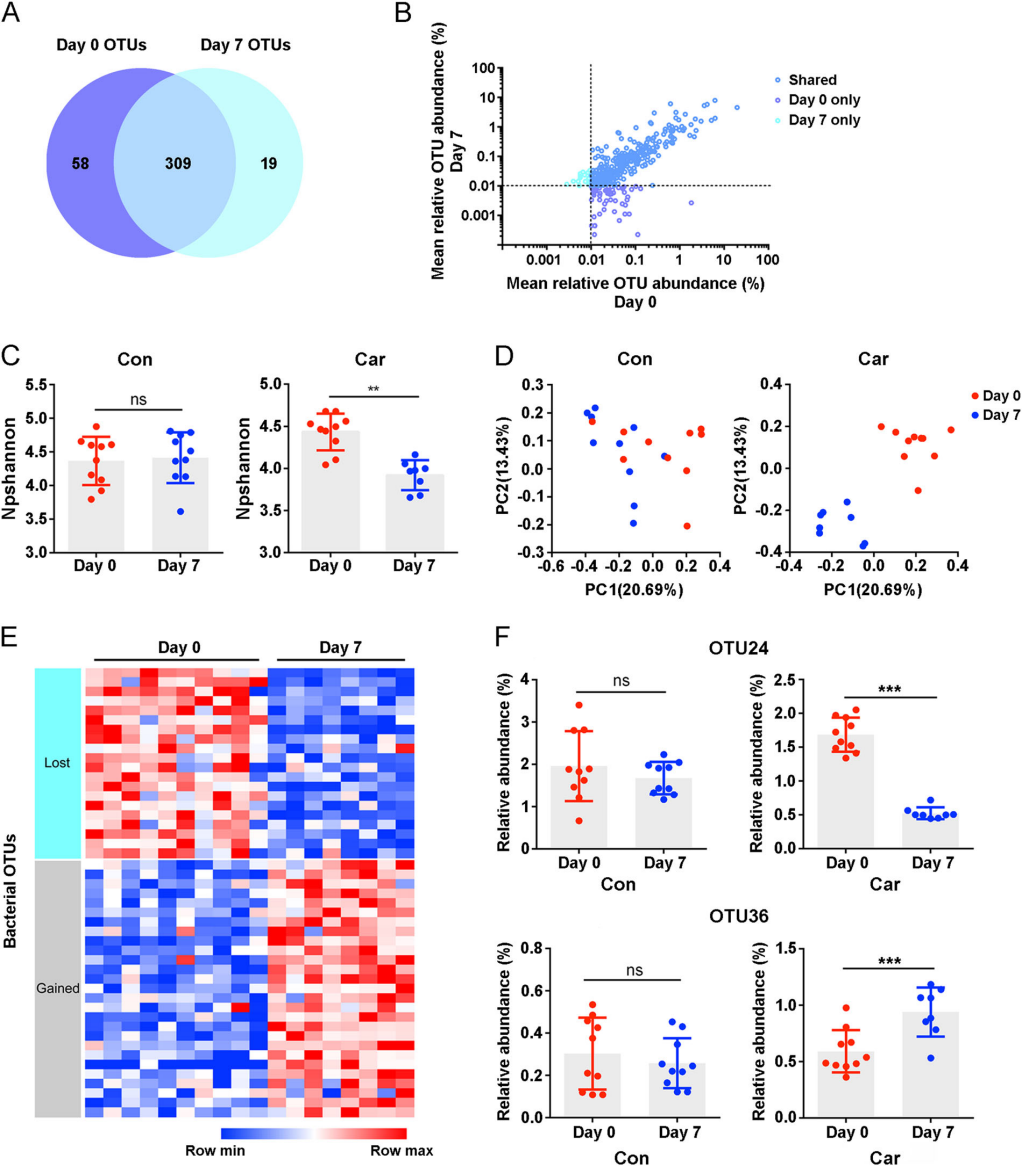
Carboplatin alters the composition of the intestinal microbiota. **a** The number of OTUs present on day 0 only (purple), day 7 only (turquoise) or shared on day 0 and day 7 (blue) in the carboplatin-treated group. **b** The mean relative abundance of OTUs present in mice before and after treatment with carboplatin. **c** Alpha diversity and **d** principal component analysis (PCA) of the gut microbiota in carboplatin-treated and control mice on day 0 and day 7. **e** Heatmap of OTUs of gut microbiota in carboplatin-treated mice. **f** Relative abundance of examples of OTUs in carboplatin-treated and control mice. Day 0 (*n* = 10); day 7 (*n* = 8). OTUs, operational taxonomic units. *p < 0.05, **p < 0.01, ***p < 0.001

Carboplatin treatment altered the microbial population in mice, as shown in Fig. 3a, b. Although most of the OTUs were shared on day 0 and day 7, 58 OTUs were lost and 19 unique OTUs were presented after treatment with carboplatin (day 7), and the total number of OTUs decreased. We next analysed the alpha diversity of bacteria in both the carboplatin-treated and control groups, and we found that the diversity of the total microbiota was significantly reduced in the carboplatin-treated group, whereas no significant change was observed in control group over time (Fig. 3c). In addition, principal component analysis (PCA) showed that the composition of the gut microbiota was distinctly altered in mice after carboplatin treatment (day 7) compared with before carboplatin treatment (day 0) (Fig. 3d). In contrast, the composition of the gut microbiota between day 7 and day 0 showed little difference in control mice (Fig. 3d). Furthermore, the markedly altered components of the gut microbiota were estimated by comparing the carboplatin-induced discrimination of normalised OTUs in mice. The OTUs of the different microbiota were decreased or increased during carboplatin treatment, as illustrated in the form of a heatmap (Fig. 3e). For example, the relative abundance of OTU24 was significantly decreased, while OTU36 was dramatically increased (Fig. 3f). Both of these OTUs showed no significant changes between day 0 and day 7 in the control group. Together, these data indicate that carboplatin treatment can profoundly alter the diversity and composition of the gut microbiota in mice.

### Identification of key microbial species correlate with intestinal mucositis

**Fig. 4:**
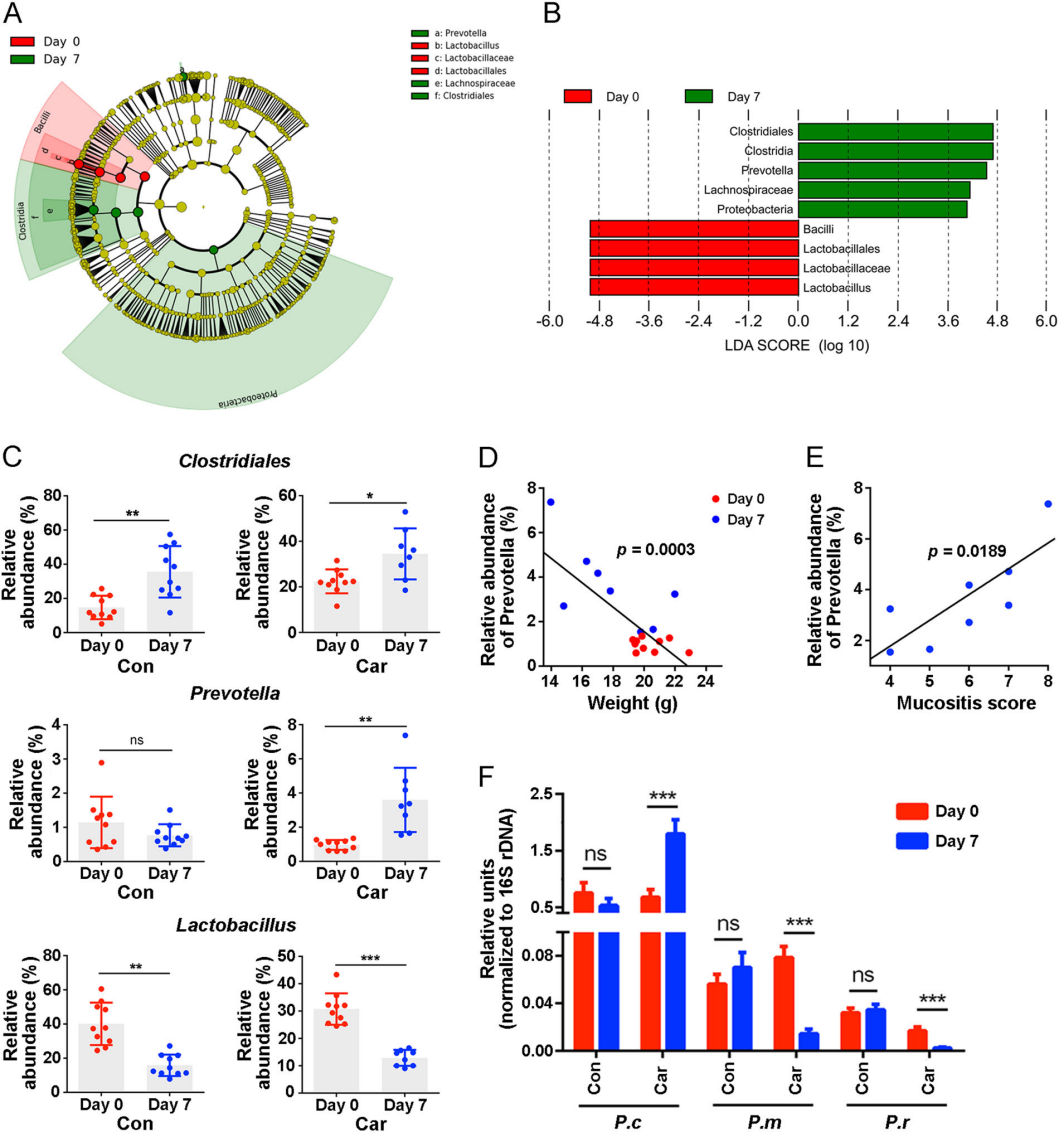
*Prevotella* is associated with carboplatin-induced intestinal mucositis. **a** LEfSe analysis of the differentially abundant taxa on day 0 (red) and day 7 (green) in carboplatin-treated mice. **b** LDA scores computed for differentially abundant taxa in carboplatin-treated mice. Only taxa with an LDA threshold >3.6 are shown. **c** Relative abundances of differentially abundant bacteria in both the control group and carboplatin-treated group. **d** and **e** The correlation analysis of *Prevotella* with body weight **d** and severity of gut mucositis **e** in carboplatin-treated mice. **f** Changes in three *Prevotella* species in mice treated with or without carboplatin. *P.c*, *Prevotella copri*; *P.m*, *Prevotella melaninogenica*; *P.r*, *Prevotella ruminicola*; ns, not significant. Data are shown as the mean ± SEM. *p *<* 0.05, **p *<* 0.01, ***p *<* 0.001

To identify the most differentially abundant bacteria in carboplatin-treated mice over time, we performed LDA effect size (LEfSe) analysis and selected biomarkers with LDA scores >4. We found that *Lactobacillus* was more abundant before carboplatin administration, while *Clostridia* and *Prevotella* were significantly enriched on day 7 (Figs. 4a, b). We further analysed the relative abundance of these bacteria in both the control group and carboplatin treatment group. The results showed that the changes in abundances of *Clostridium* and *Lactobacillus* in the carboplatin-treated group were consistent with those in the control group (Fig. 4c). However, only *Prevotella* was significantly enriched in the carboplatin-treated group, and no significant changes were observed in the control group. These results suggest that *Prevotella* was the most differentially abundant bacteria after carboplatin treatment compared with the control group.

We next investigated the relationship between the amount of *Prevotella* and intestinal mucositis. The amount of *Prevotella* was negatively associated with the weight of mice (Fig. 4d) but positively associated with the mucositis score (Fig. 4e). A large amount of *Prevotella* was strongly associated with more severe intestinal mucositis. *Prevotella* is a larger genus, but only a few species exist in the gut^32^. We performed real-time PCR to quantify the amount of specific *Prevotella* species^33^ in the faecal contents. The amount of *P. copri* distinctly increased, while that of *Prevotella melaninogenica* and *Prevotella ruminicola* significantly decreased after carboplatin but not PBS treatment (Fig. 4f). In agreement with previous reports^32,33^, *P. copri* was the most enriched bacterium among the three *Prevotella* species.

Notably, changes in *P. copri* after carboplatin treatment were consistent with the 16S rDNA sequencing results, suggesting that *P. copri* might play a role in carboplatin-induced intestinal mucositis.

### Reducing the abundance of *P. copri* relieves carboplatin-induced gut mucositis

**Fig. 5:**
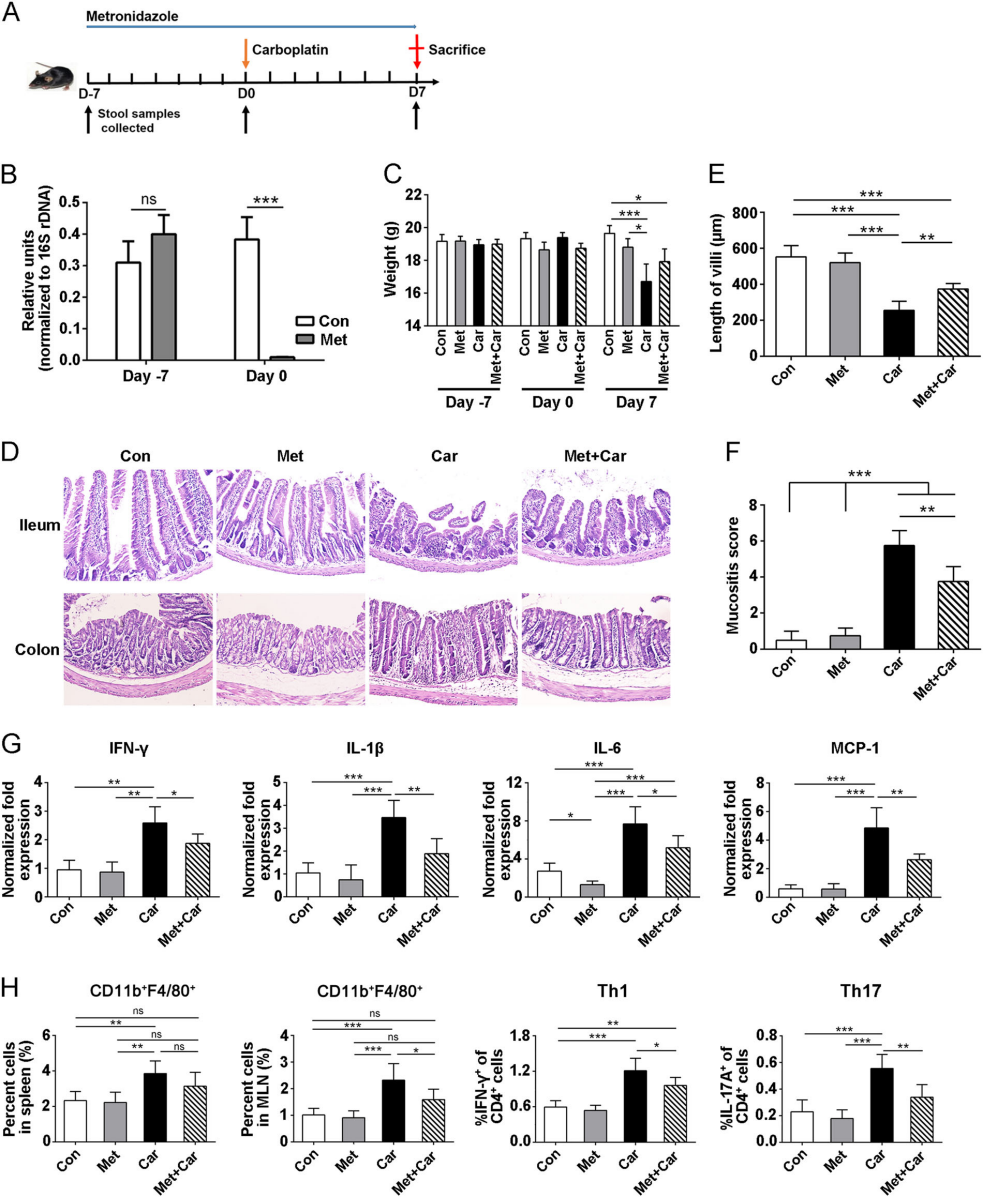
Effect of reduced *P. copri* abundance on carboplatin-induced intestinal mucositis. **a** Experimental setting. **b** Changes in *P. copri*. Mice were treated with metronidazole for 1 week, and then the relative abundance of *P. copri* in intestine was detected by real-time PCR. **c** Body weight of mice at day −7, day 0 and day 7. **d** H&E staining of ileum and colon tissues (magnification, ×200). **e** Villus length of ileum tissues. **f** Histological mucositis scores for the small intestine and colon. **g** Expression levels of IFN-γ, IL-1β, IL-6 and MCP-1 in ileum tissues of mice. **h** Percentages of CD11b^+^F4/80^+^ macrophages in spleens and MLNs. **i** Percentages of Th1 and Th17 cells in MLNs. Data are presented as the mean ± SEM. *p *<* 0.05, **p *<* 0.01, ***p *<* 0.001. Con Control; Car carboplatin; Met metronidazole

To verify the role of *P. copri* in carboplatin-induced gut mucositis, we treated mice with metronidazole for 7 days to delete the majority of bacteria, including *P. copri*, prior to the administration of carboplatin or PBS (Fig. 5a). The abundance of *P. copri* in mice was significantly reduced compared with the untreated mice (Fig. 5b). Carboplatin treatment caused a significant decrease in body weight, whereas metronidazole pretreatment followed by carboplatin treatment slightly increased the body weight of mice, although there was no significant difference between the two groups (Fig. 5c). Furthermore, in contrast to mice treated with carboplatin alone, metronidazole pretreatment resulted in a significant relief of carboplatin-induced gut mucositis, as assessed by an increase in the intestinal villus length, reduced inflammatory infiltration and a decreased mucositis score (Fig. 5d–f). The expression levels of IFN-γ, IL-β and MCP-1 in the Met mice were similar to those in the Con group, except for the downregulation of IL-6 expression (Fig. 5g). Compared with the Car group, the expression of these four pro-inflammatory factors was significantly decreased in the Met + Car group, but the expression was still higher than in the Con and Met groups (Fig. 5g), indicating that metronidazole treatment could attenuate carboplatin-induced intestinal inflammation. The contents of CD11b^+^F4/80^+^ cells in spleens and MLNs were decreased in Met + Car compared with Car group mice (Fig. 5h). However, treatment with metronidazole alone did not significantly affect the amount of CD11b^+^F4/80^+^ cells in spleens and MLNs. Similarly, pretreatment with metronidazole downregulated carboplatin-induced Th1 and Th17 cell levels in MLN (Fig. 5i).

### *P. copri* stimulates the inflammatory response in vitro

**Fig. 6:**
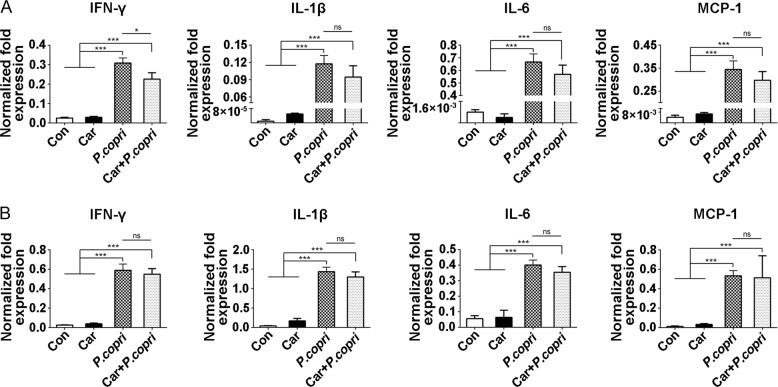
Effects of *P. copri* on the mRNA expression of IFN-γ, IL-1β, IL-6 and MCP-1 in RAW264.7 or IEC-6 cells. RAW264.7 **a** or IEC-6 **b** cells were stimulated for 4 h with medium alone, carboplatin (10 μM) or *P. copri* (multiplicity of infection = 10) in the presence or absence of carboplatin. Cells were collected and evaluated for gene expression analysis. Data are presented as the mean ± SEM. *p *<* 0.05, **p *<* 0.01, ***p *<* 0.001. Con Control; Car carboplatin

The above results confirmed that the content of *P. copri* was closely related to carboplatin-induced intestinal mucositis, and the inflammatory response decreased after clearance of *P. copri*. We next evaluated whether *P. copri* was able to induce the inflammatory response in vitro. RAW264.7 and IEC-6 cells were cultured with or without *P. copri* and carboplatin and analysed for the expression of inflammatory cytokines, such as IFN-γ, IL-1β, IL-6 and MCP-1 (Fig. 6a, b). Significant increases in the expression of these cytokines were detected in both RAW264.7 and IEC-6 treated with *P. copri* or *P. copri* *+* *car*, while carboplatin stimulation failed to upregulate the expression of inflammatory factors in these two cells. These results indicate that *P. copri*, but not carboplatin, can directly stimulate the inflammatory response in vitro.

### *P. copri* exacerbates carboplatin-associated inflammatory damage

**Fig. 7:**
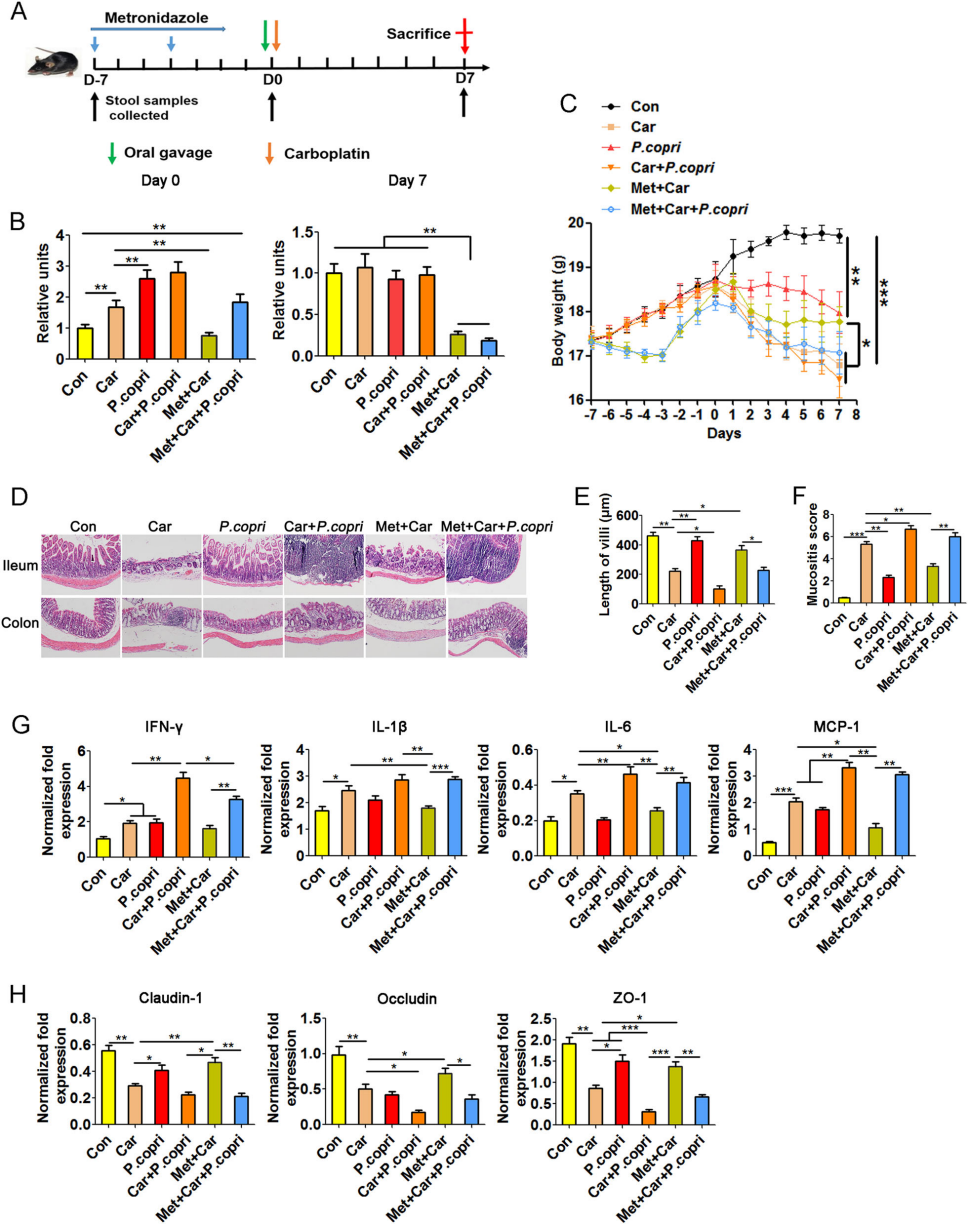
*P. copri* exacerbates carboplatin-associated inflammatory damage. **a** Experimental setting. **b** Changes in *P. copri*. Mice were treated with or without metronidazole for 5 days and then colonised with *P. copri* and treated with carboplatin on day 0. The relative abundance of *P. copri* in the intestine was detected by real-time PCR. **c** Body weight changes of the mice during the experiment. **d** H&E staining of ileum and colon tissues (magnification, ×200). **e** Villus length of the ileum tissues. **f** Histological mucositis scores of the small intestine and colon. **g** Expression levels of IFN-γ, IL-1β, IL-6 and MCP-1 in ileum tissues of mice. **h** Expression levels of claudin-1, occludin and ZO-1 in ileum tissues of mice. Data are presented as the mean ± SEM. *p *<* 0.05, **p *<* 0.01, ***p *<* 0.001. Con Control; Car carboplatin; Met metronidazole

It has been reported that *P. copri* can exacerbate inflammatory responses and promote the development of colitis^34,35^. Our results also showed that *P. copri* was positively correlated with the mucositis induced by carboplatin. To verify whether the expansion of intestinal *P. copri* led to more severe inflammatory damage, carboplatin-treated mice were colonised with *P. copri* by oral gavage (Fig. 7a). After metronidazole treatment (Day 0), the content of *P. copri* was significantly lower in the Met + Car and Met + Car + *P. copri* group than in the other three groups (Fig. 7b). At 7 days after *P. copri* gavage (Day 7), there were no significant differences in *P. copri* content between the Car + *P. copri* group and the Met + Car + *P. copri* group, but both showed significantly higher *P. copri* contents than the Car and Con groups (Fig. 7b). These results suggest that *P. copri* can robustly colonise the intestinal tract of mice.

In line with a previous report of *P. copri* exacerbating colitis in mice^35^, *P. copri*-colonised mice treated with carboplatin for 7 days presented more severe gut mucositis, as assessed by enhanced weight loss (Fig. 7c), increased epithelial damage on histological analysis (Fig. 7d), shorter intestinal villi (Fig. 7e), and a decreased mucositis score (Fig. 7f) as compared to controls treated with or without carboplatin alone. Furthermore, in contrast to mice treated with carboplatin alone, *P. copri*-colonised mice showed enhanced expression levels of inflammatory cytokines (Fig. 7g) and decreased expression levels of tight junction protein in the small intestine (Fig. 7h). These data indicate that *P. copri* can promote carboplatin-induced intestinal mucositis and enhance the destruction of the intestinal mucosal barrier system.

**Fig. 8:**
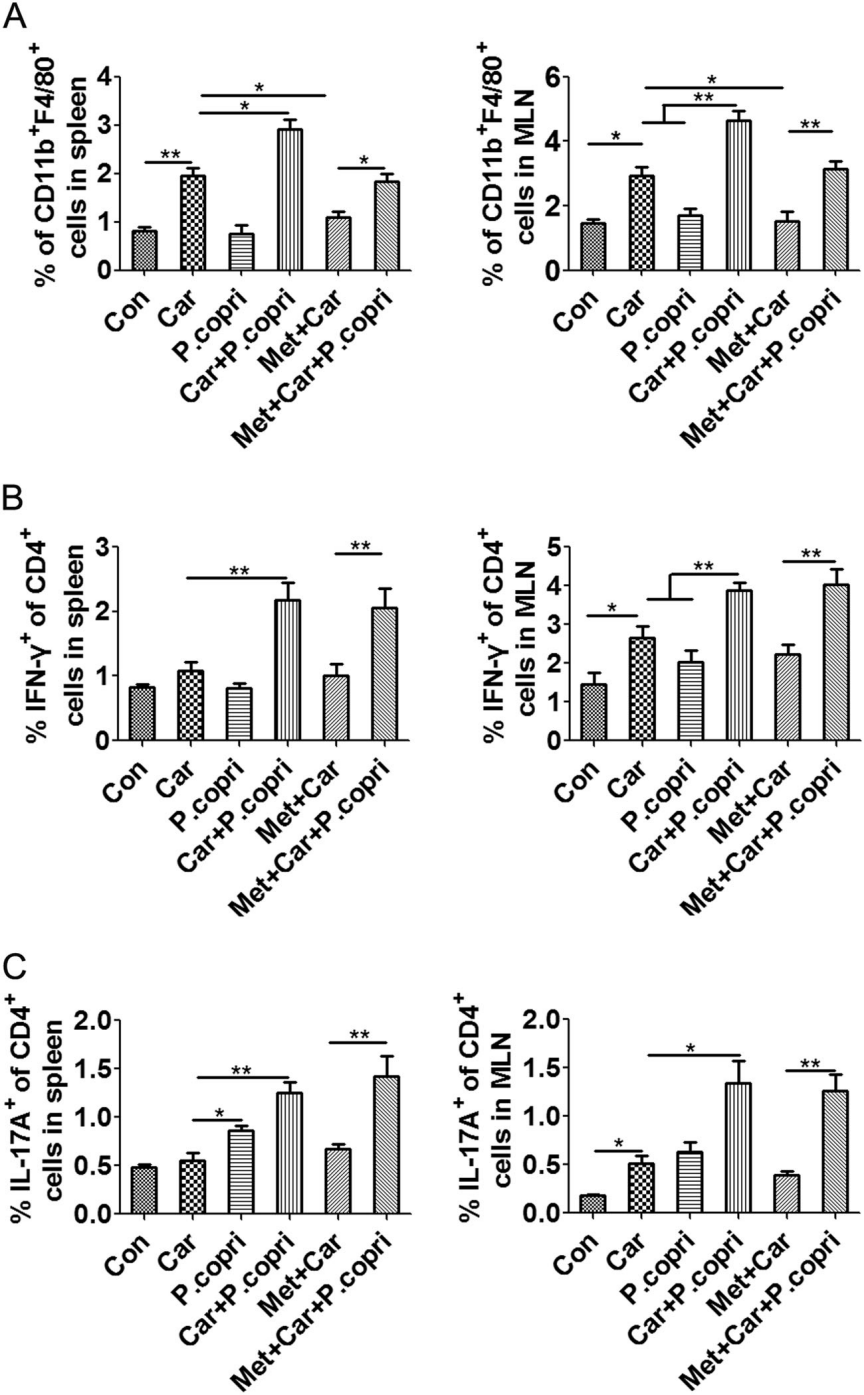
*P. copri* aggravates the carboplatin-induced macrophage imbalance and promotes the Th1 and Th17 immune response. **a** CD11b^+^F4/80^+^ macrophages in spleens and MLNs. Th1 cells **b** and Th17 cells **c** in spleens and MLNs. Data are presented as the mean ± SEM. *p *<* 0.05, **p *<* 0.01, ***p *<* 0.001. Con Control; Car carboplatin; Met metronidazole

We then used flow cytometry to analyse changes in immune cell populations at 7 days after colonisation with *P. copri*. The number of CD11b^+^F4/80^+^ cells in spleens and MLNs were markedly increased in *P. copri*-colonised mice compared with mice treated with carboplatin alone (Fig. 8a). Consistent with our previous results, carboplatin treatment alone could not significantly increase the content of Th1 and Th17 cells in the spleen (Fig. 8b, c). However, *P. copri-*colonised mice following carboplatin treatment showed significantly increased Th1 and Th17 cells in the spleen. Moreover, gavage with *P. copri* also exacerbated the increase in Th1 and Th17 cells in MLNs (Fig. 8b, c). These results suggest that *P. copri* can aggravate the carboplatin-induced macrophage imbalance and promote Th1 and Th17 immune responses, thus leading to local and systemic immune responses, which may contribute to intestinal tissue damage.

## Discussion

The toxicity caused by chemotherapy drugs seriously affects the survival rate and quality of life of cancer patients^36,37^. The gastrointestinal mucosa is a selective barrier between the body and the external environment. From the oral cavity to the rectum, cytotoxic drug treatment can lead to mucosal dysfunction and destruction, and its pathological processes include epithelial and endothelial cell death and activation of the mucosal immune system^38–40^. There are currently no successful therapies for the prevention or treatment of mucositis. Recent studies have shown that the gut microbiota can regulate the therapeutic effects of anticancer chemotherapeutic drugs, but little is known about the regulation of gut microbiota in the toxic effects of chemotherapeutic drugs.

The intestinal mucosa is the first barrier to protect the body against pathogens. Gut microbiota live on a mucus layer formed by secretion of the intestinal mucosa^7^. The gut microbiota can regulate mucosal barrier function, mucosal immune homoeostasis, prevent pathogen infection, digestion of dietary fibre, vitamin synthesis and metabolism^41^. Our study suggested that *P. copri* might be involved in carboplatin-induced mucositis. A previous study has reported that an elevated *P. copri* content can significantly increase the susceptibility of mice to colitis induced by glucan sulfate and that *P. copri* is associated with the occurrence of rheumatoid arthritis^35^. Other studies have indicated that *P. copri* may help drive chronic inflammation in HIV-infected people^42,43^. All of these studies have shown a strong relationship between *P. copri* and inflammation.

We decreased the *P. copri* abundance with metronidazole in mice and found that carboplatin-induced intestinal mucosal damage and inflammatory responses were alleviated. However, metronidazole is a broad-spectrum antibiotic that is mainly used for anaerobic bacteria, it not only removes *P. copri* but also decreases the content of many other gut microbes, the role of these microbes in the process of carboplatin-induced mucositis cannot be ruled out. Therefore, we designed additional experiments to study the effects of *P. copri* on mucositis and the results indicated that the supplementation of *P. copri* could significantly aggravate the intestinal tissue damage and inflammation induced by carboplatin. The detection of claudin-1, occludin and ZO-1 in the intestinal tissues indicated that the intestinal mucosal barrier was severely damaged. These results demonstrate that *P. copri* promotes carboplatin-induced intestinal mucositis.

The microbial community in the intestine is a large and complex system, and its steady state is related to the normal digestion, metabolism, and immune regulation of the host. The occurrence of intestinal mucositis may not be limited to the influence of a single strain, involving other strains or metabolites to form a network-like regulatory mechanism. The relationship between other strains and carboplatin-induced intestinal mucositis requires further research.

James M. Kinross et al. described the mechanism by which the gut microbiota regulates the efficacy of anticancer drugs, called the “TIMER” framework: translocation, immune regulation, metabolism, enzymatic degradation, and reduced diversity^30^. Translocation refers to the process of symbiotic or pathogenic bacteria passing through the intestinal mucosal barrier into the body system environment^44^, which may cause sepsis and/or affect the efficacy of chemotherapy. A previous study has reported that cyclophosphamide leads to destruction of intestinal structure, accompanied by the translocation of symbiotic bacteria to secondary lymphoid organs in mice^18^. The entry of specific Gram-positive bacteria into the lymphoid organs stimulated Th1 and Th17 cell responses, and the antitumour effect of cyclophosphamide was reduced after the treatment of mice with antibiotics^18^. These data suggest that the translocation of intestinal microbes can induce the body’s immune response and affect the efficacy of the drug. In the present study, carboplatin also caused atrophy of the small intestine, necrosis of intestinal epithelial cells, and destruction of the intestinal mucosal barrier. Thus, we speculated that carboplatin induced intestinal microbial translocation, and the bacteria migrated to the secondary lymphoid organs and triggered the body’s immune response. Our results showed that CD11b^+^F4/80^+^ macrophages (in the spleen and MLNs) and Th cells (Th1 and Th17 in MLNs) were significantly increased after carboplatin treatment, which might indirectly validate this hypothesis.

In vitro, we demonstrated that *P. copri*, but not carboplatin, directly promoted the upregulation of inflammatory cytokines. This finding suggested that carboplatin had no direct pro-inflammatory effect. Therefore, in addition to the direct toxic effect of carboplatin on intestinal mucosal cells, the inflammatory stimulation of *P. copri* and the local and systemic immune responses induced by the possible translocation of *P. copri* aggravated the development of mucositis. Although there are interesting results revealed by this study, there is also limitation. Carboplatin is a widely used chemotherapy drug in cancer, that is also related to gut microbiota changes. We should conduct more experiments to verify the relationship between gut microbiota and carboplatin treatment in cancer models not limited to normal mouse. Taken together, our data suggest that the regulation of gut microbes may represent a new way to reduce chemotherapy-associated intestinal toxicity.

## Supplementary information

supplementary information

Figure S1

Figure S2

Figure S3

supplementary table 1
