# Supplementary material for: Prevotella copri is associated with carboplatin-induced gut toxicity
Source: Cell Death Dis. 2019 Sep 26;10(10):714. doi: 10.1038/s41419-019-1963-9 (PMC6763498; doi:10.1038/s41419-019-1963-9)
Supplement: Supplementary file 1 — supplementary information [file 41419_2019_1963_MOESM1_ESM.docx]

**Supplementary information**

**Figure S1. Immunofluorescence staining of ileum and colon tissues.** Blue represents the nucleus, and red represents CD3^+^ cells (magnification, ×200).

**Figure S2. Expression levels of IL-17A in ileum tissues of mice after treatment with carboplatin or saline. Data are presented as the mean ± SEM. **** *p* < 0.01.

**Figure S3. Percentages of dendritic cells (DCs) in spleens and MLNs of mice treated with carboplatin or saline by flow cytometry.**

**Supplementary table 1. The list of abundance of OTUs.**
