# Supplementary figures and images for: Prevotella copri is associated with carboplatin-induced gut toxicity
Source: Cell Death Dis. 2019 Sep 26;10(10):714. doi: 10.1038/s41419-019-1963-9 (PMC6763498; doi:10.1038/s41419-019-1963-9)

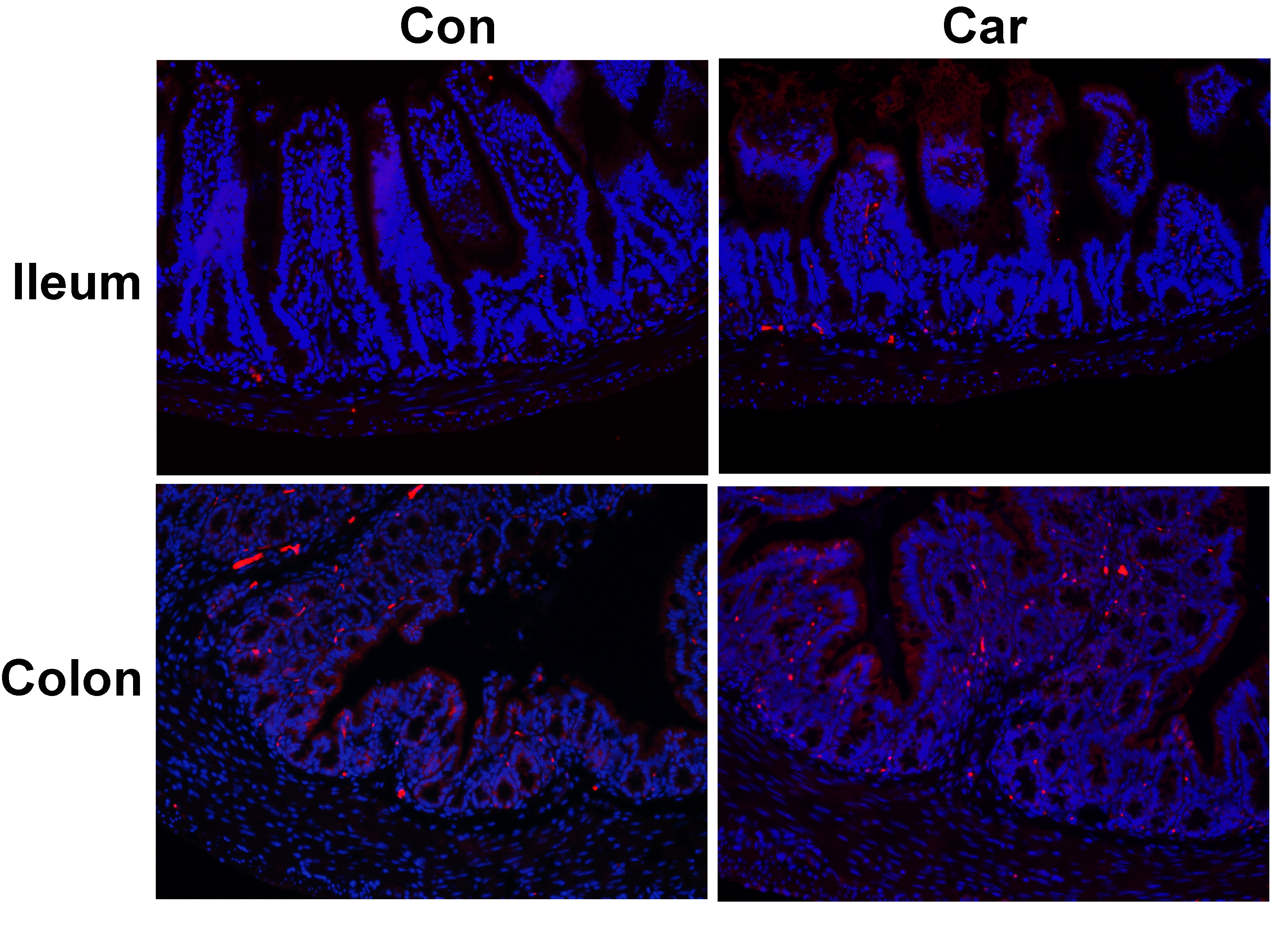

Supplement: Supplementary file 2 — Figure S1 [file 41419_2019_1963_MOESM2_ESM.tif]

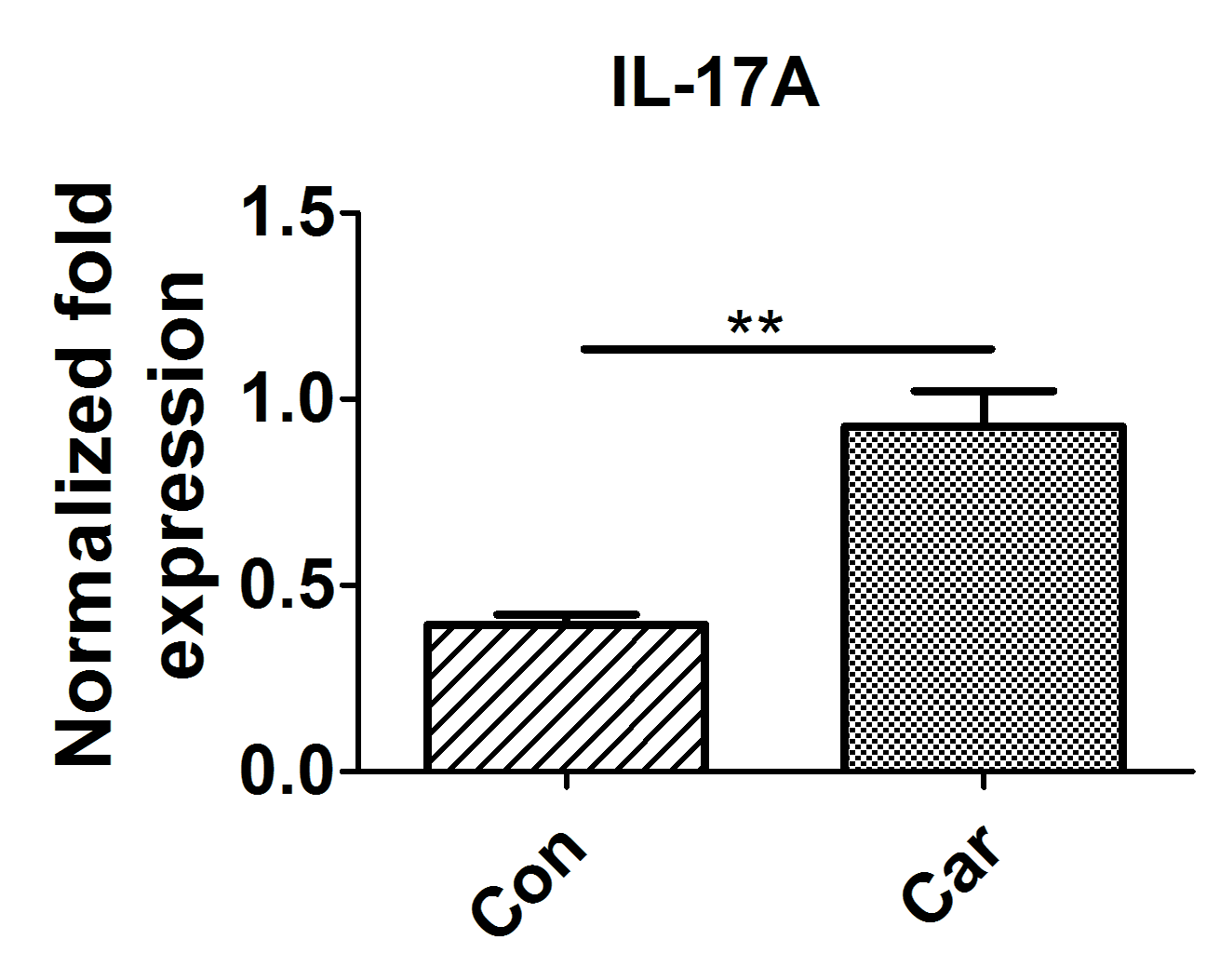

Supplement: Supplementary file 3 — Figure S2 [file 41419_2019_1963_MOESM3_ESM.tif]

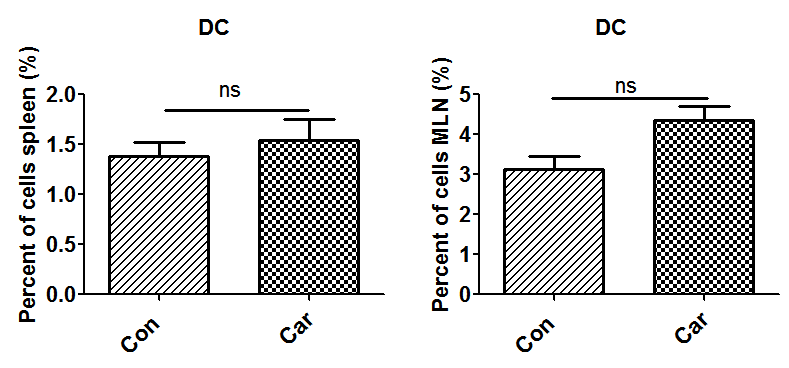

Supplement: Supplementary file 4 — Figure S3 [file 41419_2019_1963_MOESM4_ESM.tif]
